# Post-translational formation of hypusine in eIF5A: implications in human neurodevelopment

**DOI:** 10.1007/s00726-021-03023-6

**Published:** 2021-07-17

**Authors:** Myung Hee Park, Rajesh Kumar Kar, Siddharth Banka, Alban Ziegler, Wendy K. Chung

**Affiliations:** 1 Molecular and Cellular Biochemistry Section, NIDCR, National Institutes of Health, Bethesda, MD 20892 USA; 2 Division of Evolution and Genomic Sciences, School of Biological Sciences, Faculty of Biology, Medicine and Health, University of Manchester, Manchester, UK; 3 Manchester Centre for Genomic Medicine, St Mary’s Hospital, Manchester University NHS Foundation Trust, Health Innovation Manchester, Manchester, M13 9WL UK; 4 Department of Genetics, University of Angers, Angers, France; 5 Columbia University, New York, NY USA

**Keywords:** eIF5A, Hypusine, Deoxyhypusine synthase, Deoxyhypusine hydroxylase, Post-translational modification, Neurodevelopment, Translation

## Abstract

Hypusine [N^ε^-(4-amino-2-hydroxybutyl)lysine] is a derivative of lysine that is formed post-translationally in the eukaryotic initiation factor 5A (eIF5A). Its occurrence at a single site in one cellular protein defines hypusine synthesis as one of the most specific post-translational modifications. Synthesis of hypusine involves two enzymatic steps: first, deoxyhypusine synthase (DHPS) cleaves the 4-aminobutyl moiety of spermidine and transfers it to the ε-amino group of a specific lysine residue of the eIF5A precursor protein to form an intermediate, deoxyhypusine [N^ε^-(4-aminobutyl)lysine]. This intermediate is subsequently hydroxylated by deoxyhypusine hydroxylase (DOHH) to form hypusine in eIF5A. eIF5A, DHPS, and DOHH are highly conserved in all eukaryotes, and both enzymes exhibit a strict specificity toward eIF5A substrates. eIF5A promotes translation elongation globally by alleviating ribosome stalling and it also facilitates translation termination. Hypusine is required for the activity of eIF5A, mammalian cell proliferation, and animal development. Homozygous knockout of any of the three genes, *Eif5a, Dhps,* or *Dohh*, leads to embryonic lethality in mice. eIF5A has been implicated in various human pathological conditions. A recent genetic study reveals that heterozygous germline *EIF5A* variants cause Faundes–Banka syndrome, a craniofacial–neurodevelopmental malformations in humans. Biallelic variants of *DHPS* were identified as the genetic basis underlying a rare inherited neurodevelopmental disorder. Furthermore, biallelic *DOHH* variants also appear to be associated with neurodevelopmental disorder. The clinical phenotypes of these patients include intellectual disability, developmental delay, seizures, microcephaly, growth impairment, and/or facial dysmorphisms. Taken together, these findings underscore the importance of eIF5A and the hypusine modification pathway in neurodevelopment in humans.

## Introduction

Numerous cellular activities are regulated by post-translational modifications. Of those that do not involve peptide-bond cleavage, over 140 amino acids are generated from the 20 primary amino acids by modification of the N- or C-terminus or their side chains. The most common protein modifications include phosphorylation, acetylation, glycosylation, amidation, hydroxylation, and methylation. Protein-bound lysine alone can be biochemically converted to many derivatives by methylation, acetylation, hydroxylation, ubiquitylation, or sumoylation. Whereas most of these post-translational modifications occur on multiple proteins, there exist only a few exceptions in which it is limited to one protein, for example, hypusine formation in eIF5A, and diphthamide synthesis in eukaryotic elongation factor 2 (eEF2) (Su et al. 2013). After decades of research, the secrets of the specificity of the hypusine modification and its significance in eukaryotic life and human health are being unraveled.

**Fig. 1:**
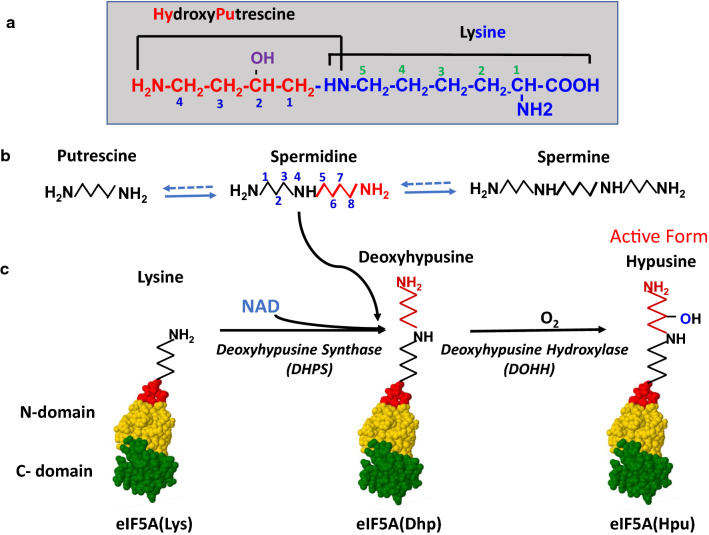
Structure of hypusine (**a**), interconversion of polyamines (**b**), and pathway of hypusine biosynthesis by two enzymatic steps (**c**). **a** Hypusine was named by combination of parts of its two structural components, hydroxyputrescine and lysine. **b** Polyamines, putrescine, spermidine, and spermine can interconvert in mammalian cells by the biosynthetic and catabolic pathways. **c** Hypusine synthesis in the eIF5A precursor occurs by two enzymatic steps catalyzed by deoxyhypusine synthase (DHPS) and deoxyhypusine hydroxylase (DOHH). N-domain of eIF5A is in yellow, C-domain in green, and the exposed, highly conserved loop containing the hypusine modification site is in red

Hypusine [N^ε^-(4-amino-2-hydroxybutyl)lysine, or (2*S*, 9*R*)-2–11-diamino-9-hydroxy-7-azaundecanoic acid] was discovered in 1971 by Shiba et al. from bovine extracts as an unusual basic amino acid (Shiba et al. 1971). It was named hypusine, based on its two structural components, hydroxyputrescine and lysine (Fig. 1a), and on the conjecture that it is formed by combination of these two components. Hypusine was found in various animal tissues (1–8 nmol/g tissue) (Nakajima et al. 1971) and also in the acid hydrolysates of animal tissue proteins (20–50 nmol/g protein) (Imaoka and Nakajima 1973). Insights into its biosynthesis and biological significance were gained nearly a decade later when a single radiolabeled protein was identified in human peripheral blood lymphocytes cultured with radioactive putrescine or spermidine (Park et al. 1981). Addition of either [1,4-^3^H]putrescine or [1,8-^3^H]spermidine to the culture medium led to the labeling of the protein, as putrescine is converted to spermidine and spermine by the polyamine biosynthesis pathway in cells (Fig. 1b). The radioa^1^ctive component of the protein was identified as hypusine, synthesized by a thus far unknown post-translational modification reaction, involving the polyamine, spermidine, and the two enzymes, deoxyhypusine synthase (DHPS) and deoxyhypusine hydroxylase (DOHH) (Park et al. 2010; Park and Wolff 2018; Wolff et al. 2007). The hypusine-containing protein was later identified as eukaryotic translation initiation factor 4D^1^ (footnote, eIF-4D, current nomenclature eIF5A) (Cooper et al. 1983).

eIF5A was initially isolated in 1976 from rabbit reticulocyte lysates (Kemper et al. 1976), as a factor that promotes the synthesis of methionyl puromycin, a model translation initiation assay and it was classified as an initiation factor and named IF-M2Bα^1^ (nomenclature at the time of its isolation) based on this activity. Later, hypusine was shown to be required for the activity of this factor in this assay (Smit-McBride et al. 1989; Park 1989). It has taken decades of seminal research from several laboratories to reach the current status of understanding of the modes of eIF5A action in translation and its significance in cell growth, animal development, and human health.

The hypusine modification is also unique in that the aminobutyl portion of the polyamine spermidine becomes covalently bound to the eIF5A precursor protein. Polyamines (putrescine, spermidine, and spermine, Fig. 1b) are organic polycations and they regulate numerous cellular activities through their interaction with nucleic acids, proteins, and phospholipids (Pegg and Casero 2011; Pegg 2016; Igarashi and Kashiwagi 2015). Polyamine homeostasis is tightly regulated by a complex network of feedback mechanisms at the transcriptional, translational and post-translational levels. Although polyamines are ubiquitous and vital in all eukaryotes, the molecular basis of their requirement is not fully understood. In the cell, the majority of polyamine content is bound to RNA and they regulate translation as polycations (Igarashi and Kashiwagi 2015; Mandal et al. 2013). The requirement of spermidine as a precursor of hypusine in the essential factor eIF5A defines a critical function of polyamines in the yeast *S. cerevisiae* growth and survival, even though only a very small fraction of cellular polyamines (1–2%) is normally used for hypusine synthesis (Chattopadhyay et al. 2008). The hypusine synthesis was also recognized as the critical function of polyamines in mammalian cell growth (Hyvönen et al. 2007; Byers et al. 1992).

eIF5A and its hypusine modification have been implicated in various human pathological conditions including cancer (Nakanishi and Cleveland 2016), diabetes (Maier et al. 2010), inflammation (Maier et al. 2010), and retroviral infections (Olsen and Connor 2017). However, these topics will not be covered in this minireview. Only recently, certain rare genetic disorders have been attributed to changes in eIF5A and the hypusine modification pathway. This connection of eIF5A to human health has been brought to light by the identification of germline variants of *EIF5A, DHPS,* or *DOHH*. Recently, de novo heterozygous *EIF5A* variants have been associated with an autosomal dominant disorder, Faundes–Banka syndrome that is characterized by developmental delay, intellectual disability, microcephaly, micrognathia, or craniofacial dysmorphism (Faundes et al. 2021). Rare biallelic pathogenic variants in *DHPS* have been included global developmental delay, intellectual disability, and seizures. Moreover, recessive rare variants of *DOHH* associated with neurodevelopmental disorder (Ziegler et al., unpublished results) have also been identified. In this review, we will discuss the biochemistry of the hypusine modification, the role of hypusine in eukaryotic cell proliferation and animal development, the mode of action of eIF5A in translation, and the importance of eIF5A and its hypusine modification pathway in neurodevelopment in humans.

### Post-translational synthesis of hypusine in eIF5A by two enzymatic steps

Hypusine is formed only post-translationally and there is no known pathway of its synthesis as a free amino acid. The free hypusine detected in the soluble extract of animal tissues (Nakajima et al. 1971; Shiba et al. 1971) was most likely generated from proteolytic degradation of eIF5A. After determination of the direct polyamine precursor of hypusine as spermidine, among the three polyamines (Park et al. 1981) (Fig. 1b), it seemed logical to assume the formation of hypusine by conjugation of a four-carbon moiety of spermidine with the side chain of lysine. However, it was not clear whether the 4-aminobutyl moiety or the 1,4-diaminobutane moiety of spermidine was transferred to lysine residue to form the hypusine residue. To distinguish between these two possibilities, the source of the secondary amino group of hypusine was determined by culturing Chinese hamster ovary (CHO) cells in medium-containing [ε-^15^ N]lysine or [4-^15^ N]spermidine and by mass spec analysis of hypusine isolated from acid hydrolysates of cellular proteins (Park et al. 1984). Hypusine enriched in ^15^N was obtained from cells cultured with [ε-^15^N]lysine, but not from those cultured with [4-^15^N]spermidine, indicating the transfer of the 4-aminobutyl moiety of spermidine during deoxyhypusine synthesis.

It was also not clear whether the hydroxylation on the hypusine side chain occurs prior to or after the transfer of the aminobutyl moiety from spermidine. As many protein hydroxylases such as lysyl- or prolyl-hydroxylases are iron-dependent enzymes, the effect of an iron chelator, α, α-dipyridyl, on hypusine synthesis was examined. Indeed, when CHO cells were cultured with radioactive spermidine or putrescine in the presence of the metal chelator, the peak of radioactive hypusine was decreased and a new radioactive peak close to the hypusine peak appeared upon ion-exchange chromatographic separation of the protein hydrolysates of the cells (Park et al. 1982). This new component was identified as the unhydroxylated form of hypusine, deoxyhypusine (N^ε^-(4-aminobutyl)lysine). The deoxyhypusine-containing eIF5A could be converted to the hypusine form in cells upon incubation in the chelator-free medium and also in cell-free lysate, providing solid evidence that the biosynthesis of hypusine occurs by way of the two enzymatic steps (Park et al. 1982). The two enzymes were named as deoxyhypusine synthase (DHS or DHPS) and deoxyhypusine hydroxylase (DOHH) (Fig. 1c).

It took years of research efforts to develop an efficient in vitro assay for DHPS. First of all, no/little labeling of eIF5A protein could be detected upon incubation of mammalian cell or tissue extracts with [1,8-^3^H]spermidine. That is because newly translated eIF5A is efficiently modified to the hypusine form in cells (Park 1987) and there is no pool of accumulated eIF5A precursor. Thus, depletion of cellular spermidine by the use of α-difluoromethylornithine (DFMO) (Park 1988) (the inhibitor of ornithine decarboxylase, the first step enzyme in polyamine synthesis) was necessary to accumulate the unhypusinated eIF5A precursors which was used as the protein substrate for DHPS (Park and Wolff 1988). It was reported that the in vitro DHPS reaction was optimum at pH 9.5, like other polyamine oxidase reactions (Murphey and Gerner 1987) and that the DHPS reaction was stimulated by the addition of NAD (Tao and Chen 1995b). Thus, development of a sensitive DHPS assay accelerated the purification of the enzyme from rat testis and the *Neurospora* (Wolff et al. 1995; Tao and Chen 1995b), and the cDNAs cloning from human, *S. cerevisiae* and *Neurospora crassa* (Tao and Chen 1995a; Kang et al. 1995; Joe et al. 1995) (a single DHPS gene exists in each of these species). The recombinant DHPS enzymes facilitated determination of the crystal structures, the reaction mechanism, and structure function studies^2^.

### Deoxyhypusine synthase (DHPS): structure and reaction mechanism

**Fig. 2:**
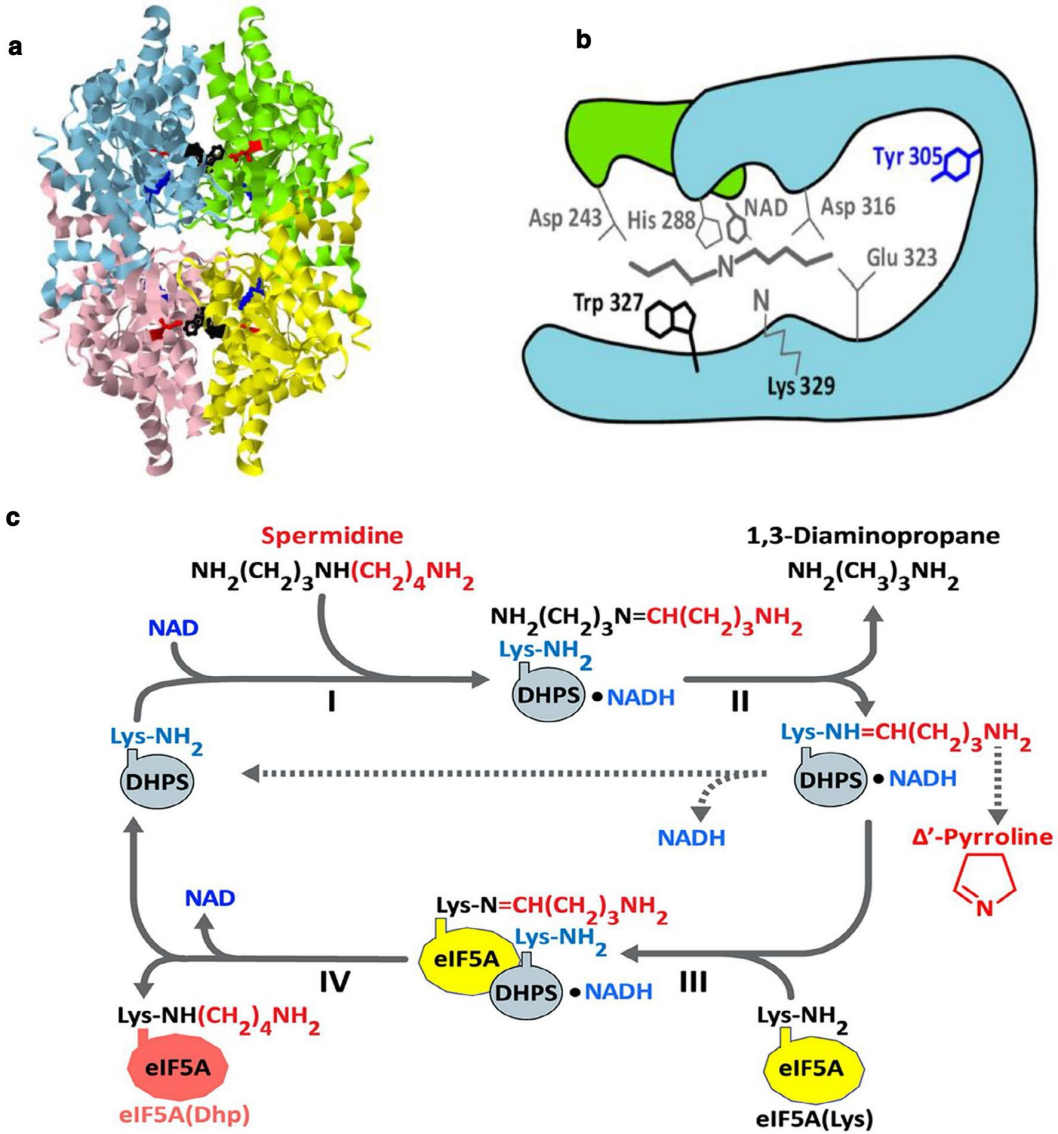
Crystal structure of DHPS (**a**), its active site (**b**), and the reaction mechanism (**c**). **a** Crystal structure of human DHPS homotetramer (PDB codes 1ROZ and 1RLZ) in complex with NAD (red) and GC7 (Umland et al. 2004). **b** A diagram of the active site of human enzyme showing the amino acid residues critical in catalysis (Lys329 and His288) and binding of spermidine (Asp243, Asp316, Glu323, and Trp327) (Lee et al. 2001). **c** Mechanism of DHPS reaction (Wolff et al. 1997)

DHPS is an NAD-dependent, tetrameric enzyme consisting of four identical subunits (~ 40 kDa) (Fig. 2a) with its active sites formed at the interface of two subunits. The crystal structures of the enzyme in complex with NAD (Liao et al. 1998) and in complex with NAD and the inhibitor and spermidine analog, N^1^-monoguanyl 1,7-diaminoheptane (GC7) (Umland et al. 2004) disclosed the amino acid residues involved in the binding of NAD and spermidine (Fig. 2b). The importance of each of the active site residues was confirmed by alanine substitution (Lee et al. 2001). The narrow groove of spermidine-binding sites consists of acidic residues, Asp243, Asp316, and Glu323 that bind one of the two terminal primary amino groups of spermidine separated by 7–8 methylene chains. The active site topology reveals the narrow specificity toward spermidine. Several spermidine analogs with two basic groups separated by 7–8 carbon chains and without a bulky substitution on the methylene chain or on secondary amino group were found to be strong inhibitors of DHPS. Among these, GC7 is the most potent inhibitor in vitro (Jakus et al. 1993), and in cultured mammalian cells when used as a single agent (Park et al. 1994) or in combination with DFMO (Schultz et al. 2018) and it exhibited antitumor activity in mice (Jasiulionis et al. 2007).

The synthesis of deoxyhypusine in eIF5A is a complex reaction occurring in four substeps (Fig. 2c): (i) NAD-dependent dehydrogenation of spermidine to generate enzyme-bound dehydrospermidine and NADH, (ii) cleavage of dehydrospermidine and transfer of the butylimine to the active site lysine (K329 in human enzyme) to form an enzyme-butylimine intermediate with the release of 1,3-diaminopropane (1,3-DAP), (iii) transfer of the butylimine moiety to the ε-amino group of a specific lysine (K50 in human protein) of eIF5A, and iv) reduction of the eIF5A-butylimine intermediate by the enzyme-bound NADH to form the deoxyhypusine residue (Wolff et al. 1997). If eIF5A(Lys)^2^ is omitted from the reaction mixture, the enzyme-butylimine intermediate accumulates, butylimine is cleaved off as ∆^1^-pyrroline and NADH is released from the enzyme and the recycled enzyme undergoes another abortive cycle of spermidine cleavage (dotted lines of half reaction). The active site residue involved in the transfer reaction was identified as Lys329 (in the human enzyme) by trapping it into a stable adduct by reduction of a reaction mixture lacking eIF5A(Lys) with NaBH_3_CN (Wolff et al. 1997). In the step iii, when eIF5A(Lys) is omitted, putrescine can also act as an acceptor of the butylimine moiety to generate homospermidine, although the Km value of putrescine (~ 1.12 mM) is much higher than that of eIF5A(Lys) (1.5 mM) (Park et al. 2003).

Apparently, the DHPS reaction can be reversed; when the radiolabeled eIF5A(Dhp) was incubated with NAD, 1,3-DAP, and DHPS, it was converted back to eIF5A(Lys) and radiolabeled spermidine was generated (Park et al. 2003). The reversal reaction proceeds through the same enzyme-imine intermediate, but in the opposite direction (Fig. 2c, steps IV to I). However, eIF5A(Hpu) was not converted back to eIF5A(Lys) when it was incubated with DHPS, NAD, and 1,3-DAP, suggesting that the presence of the OH group on the hypusine side chain prevents deoxyhypusine synthase-mediated reversal. The fact that DHPS is capable of catalyzing the reverse reaction under a specific, artificial, forced in vitro reaction condition [containing high concentration of an alternate acceptor of butylamine moiety, 1,3-DAP or putrescine in the absence of the natural acceptor, eIF5A(Lys)] should not be interpreted that the reversal reaction or synthesis of homospermidine occurs freely in vivo. Under normal physiological conditions, the accumulation of 1,3-DAP or homospermidine is not detectable in mammalian cells, as eIF5A(Lys) is by far the preferred acceptor of butylamine moiety favoring deoxyhypusine synthesis. Stable accumulation of eIF5A(Dhp) was observed in metal chelator treated cells (Park et al. 1982) or in the yeast DOHH-null strain (Park et al. 2006), without its back conversion to eIF5A(Lys).

### Deoxyhypusine hydroxylase (DOHH): cloning, structure, and reaction mechanism

DOHH is a single gene product and it catalyzes a stereospecific hydroxylation at C2 of the deoxyhypusine side chain (Fig. 1c). The in vitro enzyme assay measures the conversion of radiolabeled eIF5A(Dhp)^2^ (isolated from CHO cells cultured with α,α-dipyridyl and radioactive putrescine or spermidine, or that prepared from the in vitro DHPS reaction) to radiolabeled eIF5A(Hpu)^2^. Attempts to purify this enzyme from tissue extracts by conventional protein purification methods were not successful, as the enzyme activity was unstable and the cofactor requirement was unknown (Abbruzzese et al. 1986). Therefore, the *S. cerevisiae* GST-ORF expression library (Phizicky et al. 2002) was screened for DOHH activity and the *S. cerevisiae* DOHH clone, *YJR070C* and its human homolog *HLRC1* (Park et al. 2006) were identified. It turned out that *YJR070C* is identical to the gene *LIA1* that had been previously identified as a ligand of eIF5A from the yeast two hybrid screening (Thompson et al. 2003) and also the *S. pombe MMD1* gene encoding a novel, conserved protein essential for normal mitochondrial morphology and distribution (Weir and Yaffe 2004).

**Fig. 3:**
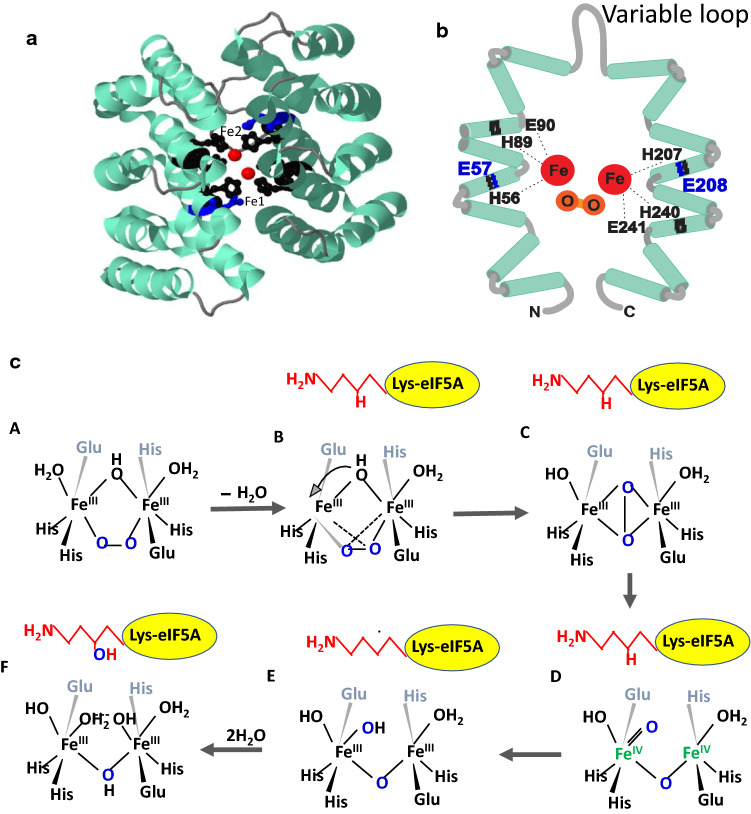
Crystal structure of DOHH (**a**), its active site (**b**), and the reaction mechanism (**c**). **a** Crystal structure of human DOHH peroxo-diiron (III) intermediate (PBD code 4D4Z) (Han et al. 2015) consisting of eight helical hairpins. **b** Active site diagram of DOHH peroxo-diiron intermediate with diiron center (red) and critical amino acid residues involved in binding diiron (black) and the protein substrate (blue). **c** Mechanism of DOHH reaction

The structure and mechanism of DOHH are distinct from the non-heme, iron-, and α*-*2-oxoglutarate-dependent dioxygenases such as lysyl- or prolyl-hydroxylases (Islam et al. 2018). Instead, the active site of DOHH resembles bacterial diiron multicomponent monooxygenases, like methane or toluene monooxygenase that uses non-heme diiron centers to activate dioxygen for the hydroxylation of hydrocarbons (Leahy et al. 2003), but its protein structure does not. DOHH has a superhelical structure consisting of eight tandem repeats of α-helical hairpins (HEAT repeats) (Fig. 3a, b) (Park et al. 2006). The iron-to-holoprotein stoichiometry of 2 was estimated for the purified recombinant holoenzyme (Kim et al. 2006). The DOHH active site contains four strictly conserved His-Glu motifs (H56-E57, H89-E90, H207-E208, and H240-E241) that are critical for its enzyme activity (Fig. 3b). Alanine substitution of each of these residues and additional conserved residues indicated the important role of the six residues His56, H89, E90, H207, H240, and E241 in anchoring the diiron center (Kim et al. 2006). The conserved residues, E57, E90, E208, E241, G65, and G214, were identified as those involved in the binding of the deoxyhypusine side chain of eIF5A(Dhp) (Kang et al. 2007). The enzyme is unique in that its diiron(III)-peroxo enzyme intermediate is exceptionally stable for days at room temperature (Vu et al. 2009). The crystal structure determined for the diiron(III)-peroxo intermediate revealed the active site residues involved in the anchoring of the diiron center and of the deoxyhypusine side chain (Han et al. 2015), that are consistent with the assignments made from mutagenesis studies. The DOHH reaction mechanism leading to the formation of the C–OH bond on C2 of the deoxyhypusine side chain has been proposed to involve the stable diiron(III)-peroxo intermediate and occur by cleavage of the O–O bond of the peroxo intermediate, abstraction of H from the target CH bond of the deoxyhypusine side chain, and rebounding of OH to complete hydroxylation (Fig. 3c) (Jasniewski et al. 2016). The enzyme is inhibited by metal chelators including α,α-dipyridyl, mimosine, ciclopirox olamine, and deferiprone.

### The specificity of DHPS and DOHH toward the eIF5A substrate

The most remarkable feature of the hypusine modification is the strict specificity toward its protein substrate eIF5A, as evidenced by radiolabeling of only eIF5A isoforms upon culture of mammalian cells with radioactive putrescine or spermidine. No other protein containing hypusine has been identified. The structural basis of the specificity was investigated using synthetic peptides and truncated polypeptides of eIF5A. No small synthetic peptides with the amino acid sequence surrounding the hypusine precursor lysine worked as a substrate for DHPS. Testing of larger polypeptides with truncations from either N- or C- terminus or from both ends of eIF5A indicated the requirement for a nearly intact N-domain of eIF5A(Lys) (aa30-90 as the minimum size) as the substrate for DHPS (Joe and Park 1994). In addition, a similar minimum size of eIF5A(Dhp) peptides (aa20-90, minimum) was required for the DOHH reaction (Wolff et al. 2007). The macromolecular interactions between eIF5A(Lys) and DHPS, and those between eIF5A(Dhp) and DOHH, required for fruitful modifications may be the basis of the extremely narrow substrate specificities.

### Role of deoxyhypusine/hypusine modification in eIF5A activity and the viability of yeast and higher eukaryotes

eIF5A is a small acidic protein with two domains, a basic N-terminal domain and an acidic C-terminal domain with an oligonucleotide-binding (OB) fold structure (Fig. 1c) (Dever et al. 2014). The amino acid sequence of eIF5A is highly conserved in eukaryotes. 3Like eIF5A, the amino acid sequences of DHPS and DOHH are also highly conserved in eukaryotes, especially at the active sites residues involved in substrate binding and catalysis (Wolff et al. 2007). The high conservation of DHPS and DOHH may have been mandated by the requirement for the macromolecular interaction between eIF5A and either of the enzymes. Hypusine synthesis occurs at a specific lysine residue (Lys50 in humans, Lys51 in yeast) on the strictly conserved, exposed loop of the N-domain (orange with red loop, Fig. 1c). Our early finding that the labeling of the hypusine-containing protein increased dramatically in mitogen-treated lymphocytes hinted at a role for this protein in cell proliferation (Park et al. 1981). Biochemical evidence for the critical role of hypusine/deoxyhypusine was obtained in vitro and in cells. The unhypusinated precursors, recombinant human eIF5A(Lys) expressed in *E. coli*, and the two forms of eIF5A precursors, eIF5A(K50) and eIF5A(AcK47/K50), isolated from spermidine depleted CHO cells were inactive in the methionyl-puromycin synthesis assay (Park 1989; Smit-McBride et al. 1989). On the other hand, eIF5A(Dhp), generated from recombinant eIF5A(Lys) by the in vitro DHPS reaction, displayed a partial activity, whereas the homodeoxyhypusine form, eIF5A(hDhp) with a sidechain one methylene longer than deoxyhypusine, did not (Park et al. 1991), suggesting a strict structural requirement for the hypusine side chain length. The in vivo evidence for the importance of the deoxyhypusine/hypusine modification was obtained in a *S. cerevisiae* strain in which its two eIF5A genes, *TIF51A* (*HYP2*) and *TIF51B* (*HYP1*), were disrupted and its growth was supported by plasmid born eIF5A. A plasmid encoding wild-type eIF5A(Lys51) supported growth of the eIF5A-null strain whereas that encoding a mutant form, eIF5A(K51R), that cannot be modified to the hypusine form, did not (Schnier et al. 1991). Furthermore, *S. cerevisiae* cells lost viability when the DHPS gene was disrupted (Sasaki et al. 1996; Park et al. 1998). Interestingly, the DOHH-null yeast strain containing only eIF5A(Dhp) but no eIF5A(Hpu) was viable (Park et al. 2006) and its growth rate was only slightly reduced compared to the wild type. This finding suggests that eIF5A(Dhp), partially active in the methionyl-puromycin synthesis assay, can support yeast growth and viability. In contrast, the maturation of eIF5A(Dhp) to eIF5A(Hpu) by DOHH appears to be crucial for the viability of multicellular eukaryotes. The ablation of DOHH expression blocked embryonic development in *C. elegance* (Sugimoto 2004), and *Drosophila* (Patel et al. 2009). Furthermore, homozygous whole-body knockout of any of the three genes, *Eif5a, Dhps* (Nishimura et al. 2012), or *Dohh* (Sievert et al. 2014), led to early embryonic lethality in mice, and the whole-body knockout of *Dhps* or *Dohh* in adult flox/flox mice using an inducible Cre expression also resulted in the growth inhibition and death in 1–5 weeks (Pällmann et al. 2015). The postnatal tissue-specific knockout of *Eif5a* or *Dhps* resulted in the inhibition of organ development (Padgett et al. 2021; Levasseur et al. 2019).

Two or more eIF5A isoforms^3^ exist in various eukaryotes. eIF5A isoforms are highly conserved and they all undergo hypusine modification. In yeast, the expression of the two eIF5A genes is regulated by oxygen; *TIF51A* is expressed under aerobic conditions and *TIF51B* under anaerobic conditions (Mehta et al. 1990). Expression of either of the two yeast or the two human eIF5A isoforms supported growth of *S. cerevisiae* (Schwelberger et al. 1993) suggesting their functional identity in yeast and the requirement for expression of only one isoform for yeast viability. However, the eIF5A isoforms may have differentiated functions in higher eukaryotes like mammals. In mammals, the main isoform, eIF5A1 (generally called eIF5A), is constitutively expressed in all cells and tissues, whereas the second isoform, eIF5A2 protein, is not normally detectable, except in certain cancer cells (Clement et al. 2006). The eIF5A2 gene resides in chromosome 3q26, a region frequently amplified in various human cancers and eIF5A2 overexpression has been associated with certain human cancers (Guan et al. 2001). Unlike eIF5A (eIF5A1), the homozygous *Eif5a*2 knockout mouse is viable, indicating that it is dispensable for normal development and viability (Pällmann et al. 2015). Apparently, the *Eif5a2* cannot replace *Eif5a* during embryonic development in mice, as the homozygous knockout of *Eif5a* is lethal (Nishimura et al. 2012).

### Mode of action of eIF5A in translation

eIF5A^1^ (initial nomenclature IF-M2Ba, then eIF-4D) is not a translation initiation factor as its name indicates, but it is a translation elongation factor. It was initially classified as a translation initiation factor, based on its stimulating activity in methionyl-puromycin synthesis (Kemper et al. 1976), a model assay for peptide-bond synthesis, that measures the transfer of methionine from the donor, initiator methionyl tRNA to the acceptor puromycin, a poor substrate for the peptidyl transferase reaction. The role of eIF5A in translation initiation was questioned, as it did not enhance any of the steps leading to the formation of the 80S initiation complex, such as binding of initiator methionyl tRNA or mRNA to ribosome (Benne and Hershey 1978). Moreover, eIF5A enhanced methionyl-puromycin synthesis when added to a preformed 80S initiation complex, whereas other initiation factors did not. Definitive evidence for the role of eIF5A in translation elongation was provided by polysome profiles of *S. cerevisiae* mutants harboring temperature-sensitive eIF5A or those with eIF5A expression under an inducible promoter (Dever et al. 2014; Gregio et al. 2009; Saini et al. 2009). Polysome profiles indicated that depletion or inactivation of eIF5A resulted in the accumulation of polysomes (Saini et al. 2009; Gregio et al. 2009), similar to that observed in a translation elongation mutant. Furthermore, depletion of eIF5A in the mutants caused a significant decrease in total protein synthesis and an increase in the ribosome transit time.

**Fig. 4:**
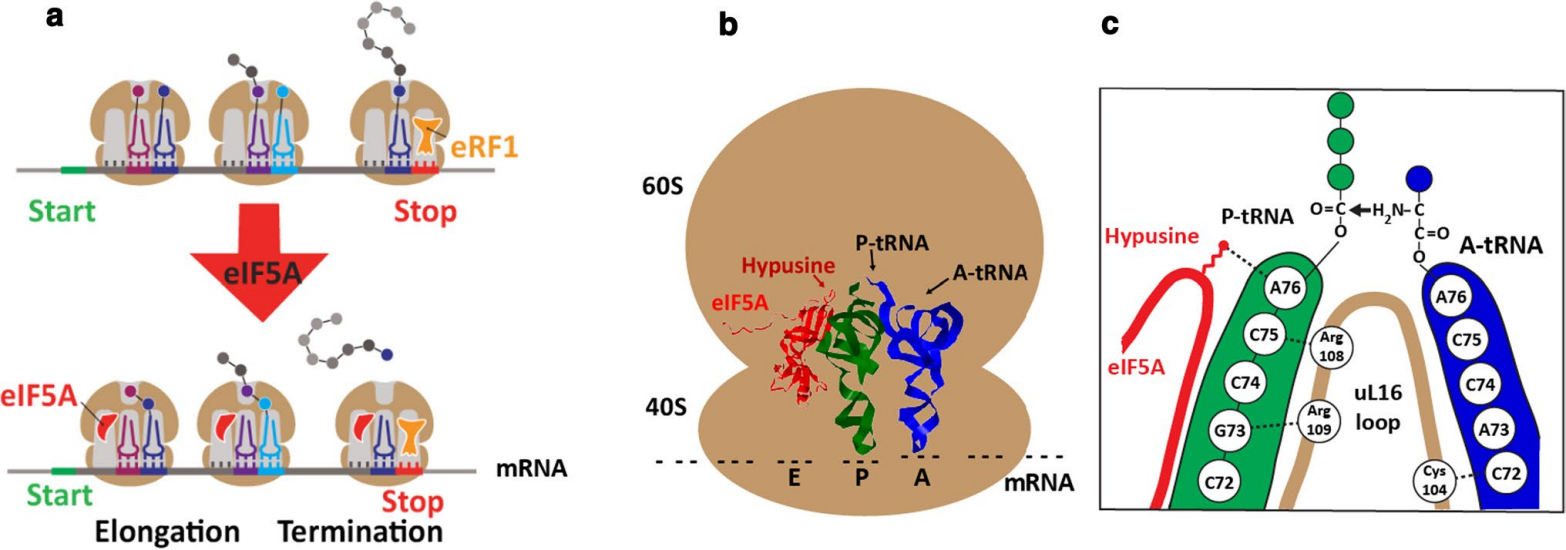
Role of eIF5A in translation elongation and termination (**a**), proposed modes of eIF5A binding to 80S ribosome (**b**), and its action in translation elongation (**c**). **a** The hypusinated eIF5A promotes peptide-bond formation between A-tRNA and P- tRNA on the 80S ribosome. It also facilitates translation termination by enhancing eRF-1-mediated hydrolysis of P-tRNA and release of the nascent peptide. (adapted from (Schuller et al. 2017)). **b** eIF5A (red;PDBcode 5GAK) is bound to the yeast ribosome at the exit tRNA site adjacent to the P-tRNA (green; PDB code 5GAK). The A-tRNA is shown in blue (PDB code 5GAK). Abbreviations: E, exit tRNA site; P, P-tRNA site; A, A-tRNA site. **c** The hypusine side chain of eIF5A (red) contacts A76 of the CCA end of P-tRNA to stabilize it and its nascent peptide chain. It also promotes interactions between the ribosomal protein uL16 with both A- and P-tRNA and thereby stimulates peptide-bond formation (modified from Schmidt et al. 2016)

During the translation elongation, peptide-bond synthesis between the aminoacyl tRNA (A-tRNA) and the peptidyl tRNA (P-tRNA) can stall at certain amino acids, such as proline or glycine that are poor substrates of the peptidyl transferase reaction. A delay in the peptidyl transferase step can cause ribosome stalling or a drop-off of the P-tRNA. Gutierrez et al. reported evidence, suggesting that eIF5A has activities in the stimulation of synthesis of proline-repeat motifs in *S. cerevisiae* and also in in vitro reconstituted peptide synthesis (Gutierrez et al. 2013). However, ribosome profiles of eIF5A-depleted *S. cerevisiae* cells revealed abundant stalling at a wide spectrum of amino acid triplets, not limited to proline-repeat sites, including those containing proline, aspartic acid, glycine, alanine, valine, and isoleucine (Schuller et al. 2017). eIF5A stimulated peptide-bond formation of a widely broad combination of amino acids in an in vitro reconstituted translation system. These results support the idea that eIF5A promotes translation elongation broadly beyond the proline repeats (Fig. 4a). This view is consistent with eIF5A activity in methionyl-puromycin synthesis as an elongation factor. The ribosome profiles of eIF5A-depleted cells also showed increased ribosome accumulation at stop codons, suggesting a global defect in termination. Furthermore, eIF5A markedly enhanced the hydrolysis of the P-tRNA and the release of a nascent protein in the in vitro assay, containing the elongation complexes with [35S]-Met-Phe-Lys-tRNALys in the P site and a stop codon (UAA) in the A site, and the eukaryotic release factors 1 and 3 (eRF1:eRF3) (Fig. 4a) (Schuller et al. 2017). The presence of eIF5A in excess over the ribosome is in agreement with the notion that eIF5A contributes to most peptidyl transfer events in translation.

The cryo-electron microscopy reconstruction of yeast hypusinated eIF5A bound to yeast 80S ribosome (Schmidt et al. 2016) provided further insights into the mode of action of eIF5A in translation elongation. eIF5A is bound at the exit tRNA site, adjacent to the P-tRNA (Fig. 4b) with the hypusine side chain reaching toward the peptidyl transferase center of the ribosome. The hypusine side chain of eIF5A(Hpu) contacts A76 of of the CCA end of the P-tRNA through a hydrogen bond (Fig. 4c). This interaction stabilizes and orients the peptidyl loop to facilitate the nucleophilic attack by the A-tRNA in the A site (Fig. 4c). The hydroxyl group of the hypusine side chain can also form hydrogen bond with the phosphate backbone of A2808 of 25S rRNA. While the hydrogen bond formed by the hydroxyl group of hypusine side chain is dispensible for translation in yeast, it may be critical for the action of eIF5A in facilitating peptidyl transfer on the ribosomes of higher eukaryotes.

### Association of variants of *EIF5A*, *DHPS, *and *DOHH* in rare neurodevelopmental disorders in humans

Translational fidelity and efficiency are vital for the survival of living organisms. Errors during mRNA translation can lead to an increase in deleterious proteins, while reducing the functional proteins (Kapur and Ackerman, 2017). From whole trio exome sequencing, variants in *EIF5A, DHPS,* and *DOHH* genes were identified as the basis of certain rare neurodevelopmental disorders in humans.

**Fig. 5:**
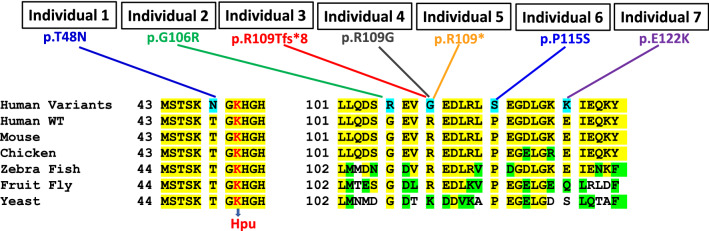
Heterozygous variants of *EIF5A* associated with a novel craniofacial neurodevelopmental disorder. The sites of missense variants (in aquablue) are indicated for each of seven patients and are located in the highly conserved region of eIF5A. The amino acid sequences from residues 43–53 and from residues 101–127 (numbering of human eIF5A) are shown. The yellow highlight indicate conservation among the six species and green highlights, conservative replacement. The totally conserved lysine that undergoes hypusine modification is indicated in red

Rare de novo heterozygous *EIF5A* variants were recently described in seven individuals (four females and three males, ages from 8 months to 18 years) with syndromic developmental delay and intellectual disability (Table 1) (Faundes et al. 2021). The condition is now designated as Faundes–Banka syndrome (OMIM 619,376). The level of developmental delay or intellectual disability was moderate in most cases. Intrauterine growth retardation was noted in three, and neonatal feeding difficulties in four individuals. Congenital cardiac anomalies were reported in three individuals. Notably, the head circumferences of the two youngest individuals were within normal ranges and all other individuals were significantly, or nearly, microcephalic. Facial dysmorphic features were variable, and included broad eyebrows, abnormal supraorbital ridges, epicanthic folds, telecanthus, thin upper lip, micrognathia, and low-set ears. All seven individuals had distinct *EIF5A* variants, which included five missense, one nonsense, and one frameshift mutations (Fig. 5). One of the missense variants resulted in the substitution at position T48, which is proximal to the K50 hypusination site. All other variants were located in the oligonucleotide-binding (OB) fold domain. Position R109 was affected in three individuals, one each with missense, nonsense, and frameshift mutation. These variants were shown to impair eIF5A function, its interaction with ribosome, and synthesis of proteins with poly-proline tracts in yeast-based assays. The severity of the in vitro phenotypes with individual variants appeared to correlate with the severity of the human phenotypes, but the sample size was too small to make reliable genotype–phenotype correlations. Treatment with spermidine showed partial rescue of the phenotype in the yeast and in morphant zebrafish models (Faundes et al. 2021). The mechanism of how spermidine rescues the phenotypes in these models remains to be investigated. Spermidine may lead to increased hypusine synthesis, although it is not a rate limiting factor under normal conditions. Alternatively, Spermidine might rescue the phenotypes by directly enhancing the efficiency and fidelity of protein synthesis (Shin et al. 2017).

**Table 1:** Phenotypes of the affected individuals with heterozygous *EIF5A* variants or biallelic *DHPS* variants

Individual	Gene	Genotype	Protein	Sex/age	Inheritance/zygosity	Clinical phenotypes
1	*EIF5A*	c.143C>A	p.T48N	F(6.9 y)	DN Het	Developmental delay, intellectual disability, facial dysmorphisms, microcephaly
2	*EIF5A*	c.316G>A	p.G106R	F(8.4y)	DN Het	Developmental delay, intellectual disability, facial dysmorphisms, microcephaly, cardiac anomalies, growth (LW, SS)
3	*EIF5A*	c.324dupA	p.R109Tfs*8	F(8.4y)	DN Het	Developmental delay, intellectual disability, facial dysmorphisms, microcephaly, cardiac anomalies
4	*EIF5A*	c.325C > G	p.R109G	M(18.3y)	DN Het	Developmental delay, intellectual disability, facial dysmorphisms, hypotonia, microcephaly
5	*EIF5A*	c.325C > T	p.R109*	M(8 mo)	DN Het	Developmental delay, intellectual disability, facial dysmorphisms, cardiac anomalies, hypotonia, growth (LW)
6	*EIF5A*	c.343C > T	p.P115S	M(4 y)	DN Het	Developmental delay, intellectual disability, facial dysmorphisms
7	*EIF5A*	c.364G > A	p.E122K	F(16.4 y)	DN Het	Developmental delay, intellectual disability, facial dysmorphisms, microcephaly, growth (SS)
8	*DHPS*	c.518A > G/c.1014 + 1G > A	p.N173S	F(9 y)	BAR Inheritance	Developmental delay, intellectual disability, facial dysmorphisms seizures, growth (LW, SS)
9	*DHPS*	c.518A > G/c.1014 + 1G > A	p.N173S	M(5 y)	BAR Inheritance	Developmental delay, intellectual disability, facial dysmorphisms, hypotonia, seizures, growth (LW, SS)
10	*DHPS*	c.518A > G/ c.912-917delTTACAT	p.N173S/ p.Y305-I306 del	F(7 y)	BAR Inheritance	Developmental delay, intellectual disability, facial dysmorphisms, microcephaly, growth (LW, SS)
11	*DHPS*	c.518A > G/c.1014 + 1G > A	p.N173S	F(8 y)	BAR Inheritance	Developmental delay, intellectual disability, facial dysmorphisms, hypotonia, seizures, microcephaly, growth (LW, SS)
12	*DHPS*	c.518A > G/c.1A > G	p.N173S/p.Met1?	F(24 y)	BAR Inheritance	Developmental delay, intellectual disability, seizures, growth (LW, SS)

*DN Het*, de novo heterozygous; *BAR*, biallelic recessive; *LW*, low weight; *SS*, short stature, *y* years

Transcripts with RefSeq ID NM_001970.5 and NM001930.3 have been used to denote the *EIF5A * and DHPS variants, respectively

**Fig. 6:**
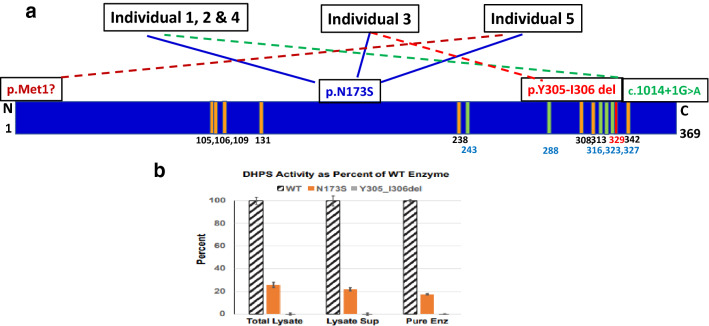
Biallelic variants of *DHPS* (NM 001,930.3) associated with rare neurodevelopmental disorder. **a** The location and the nature of variants in the five patients are indicated on the bar of DHPS sequence and are connected to each patient with solid and broken lines. Three patients (1, 2, and 4) share the same genotypes and all patients c share N173S variant. The amino acid residues involved in the binding of NAD (orange), spermidine (green), and the critical active site residue Lys 329 are indicated. **b** Reduced activity of variant DHPS enzymes from in vitro assay. The enzyme N173S is partially active with approximately 20% of the wild-type enzyme activity. The enzyme with deletion of Tyr 305-Ile306 is totally inactive

**Fig. 7:**
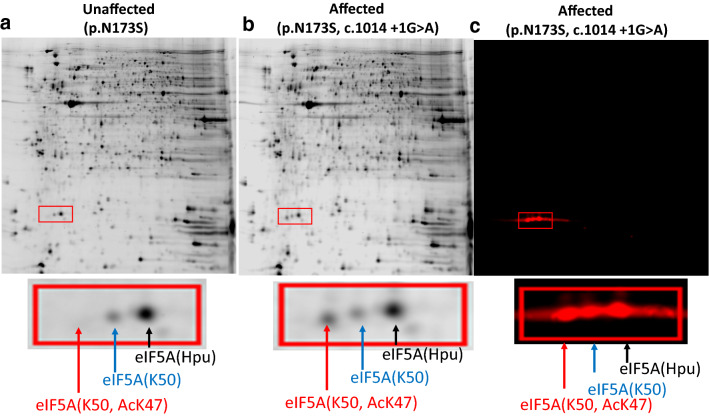
Two-dimensional gel analysis of proteins of lymphoblastoid cells derived from an affected and unaffected individuals with *DHPS* variants **a** Protein pattern of an unaffected parent with one allele variant p.N173S. **b** The protein pattern of an affected individual with biallelic variants (p.N173S, c1014 + 1G > A) that shows a decrease in the hypusinated eIF5A and the accumulation of the unhypusinated eIF5A precursors, PI (Park 1989), which was later identified as eIF5A(AcK47, K50) and PII, eIF5A(K50). **c** Western blot of cellular proteins of the affected patient in **b** with an eIF5A antibody (BD Bisciences) that recognizes all three forms of eIF5A. eIF5A(Dhp) containing deoxyhypusine residue also runs very close to eIF5A(K50), so the spots indicated as eIF5A(K50) in **a**, **b** and **c** may contain a small fraction of eIF5A(Dhp)

Rare biallelic, inherited, recurrent, pathogenic variants in *DHPS* have been identified in five affected individuals from four unrelated families and segregate with the neurodevelopmental condition in these families (Ganapathi et al. 2019) (OMIM 600944). The patients have similar neurodevelopmental features that include global developmental delay, hypotonia, and seizures (Table 1). Patients have challenges with motor coordination and balance, in part due to both hypotonia and hypertonia and spasticity. Seizure types include absence and tonic clonic seizures and seizures during sleep. Short stature, microcephaly, and mildly dysmorphic features are observed frequently but not consistently. All five affected individuals reported to date share a recurrent missense variant (c.518A > G:p.N173S) in *trans* with a likely gene disrupting variant (c.1014 + 1G > A, c.912_917delTTACAT:p.Y305_I306del, or c.1A > G:p.Met1?) (Fig. 6a). Molecular studies demonstrated that the c.1014 + 1G > A variant causes aberrant splicing. Recombinant DHPS enzyme with either the p.N173S or p.Y305_I306del variant showed reduced (20%, p.N173S) or absent (p.Y305_I306del) in vitro activity, respectively (Fig. 6b). Each affected individual contains one inactive allele (Fig. 6a, broken lines) and a partially active allele, p.N173S (Fig. 6a, solid blue line). Thus, all affected individuals have some residual DPHS activity from p.Asn173Ser, and it is likely that complete loss of activity is not compatible with life. Notably, heterozygous parents and family members are all asymptomatic, suggesting that 50% of activity would be sufficient and provides a goal for therapeutic intervention. Two-dimensional gel analysis of proteins of lymphoblastoid cells derived from unaffected (Fig. 7a) and affected individuals (Fig. 7b, c) showed a reduction in the hypusinated eIF5A, eIF5A(Hpu)^3^, and the accumulation of unhypusinated eIF5A precursors, eIF5A(K50) and eIF5A(AcK47, K50) in affected individual (Fig. 7b, c), providing the in vivo biochemical evidence of limited eIF5A hypusination in cells expressing two *DHPS* variants (Fig. 7).

Rare biallelic loss of function variants in *DOHH* were identified in an 8 year old girl presenting a severe neurodevelopmental disorder with several symptoms such as hypotonia, dysmorphic features, and microcephaly, overlapping with those found in *DHPS* and *EIF5A*-related disorders. Like the *DHPS* variants associated disorder, both parents who are heterozygous carriers are asymptomatic, suggesting that haploinsufficiency of this gene is tolerated. These DOHH variant enzymes displayed markely reduced DOHH activity in vitro. A reduction in eIF5A(Hpu) with an accumulation of unhydroxylated form, eIF5A(Dhp), was observed in the fibroblasts derived from the affected individual, suggesting that a reduction in eIF5A(Hpu) was responsible for the phenotypes. Further investigations are ongoing to determine the impact of other biallelic *DOHH* variants of unknown significance identified in a cohort of individuals with developmental delay.

## Concluding remarks

The essential feature and unique specificity of hypusine biosynthesis and the high conservation of eIF5A, DHPS, and DOHH attest to the vital importance of this post-translational modification. Starting from the discovery of a new amino acid in one specific protein, we have gained a full spectrum of knowledge on this biochemical pathway and its importance to eukaryotic life and human health. Hypusine modification occurs in eukaryotes and certain *archaea,* but not in bacteria. Yet, eIF5A is a universally conserved translation factor with structural and functional analogy with its bacterial ortholog, elongation factor P (EF-P) (Dever et al. 2014). The evolutionary progression of the essentiality and the structural stringency of eIF5A and its hypusine modification (Wolff et al. 2007) may have been dictated by an increased demands to translate complex proteins of higher eukaryotes with higher fidelity and efficiency. The variants in *EIF5A, DHPS,* and *DOHH* are believed to exert their effects through a common channel, eIF5A, either by a reduction in biologically active, hypusinated eIF5A or through impairment in eIF5A function. The fact that these variants lead to neurodevelopmental disorders suggests that, among all other organs and tissues, brain is most sensitive to a deficiency in biologically active eIF5A. The translational errors resulting from a reduction in active eIF5A may lead to an accumulation of aberrant proteins that are toxic to the neural system and impair brain function. It is not known whether neurodevelopmental features in patients with variants in *EIF5A, DHPS,* or *DOHH* are due to general effects of translational errors or due to a reduction of critical factors in brain development that are specifically dependent on eIF5A. Mouse models with a knockout of *Eif5a* or *Dhps* in a temporally and spatially specific manner in brain have been developed (Kar et al. 2021). These mice display impairment in growth, lifespan and cognitive functions, reflective of phenotypes of human patients, and may serve as useful tools in the development of chemical or biological therapeutics against neurodevelopmental disorders caused by variants of *EIF5A, DHPS,* or *DOHH*.

## Abbreviations

eIF5A: Eukaryotic initiation factor 5A
eIF5A(Lys)/eIF5A(K50): EIF5A precursor with lysine 50
eIF5A(AcK47, K50): EIF5A precursor with acetyllysine 47 and lysine 50
eIF5A(Dhp): EIF5A containing deoxyhypusine
eIF5A(Hpu): EIF5A containing hypusine
eIF5A(hDhp): EIF5A containing homodeoxyhyhypusine
DHPS: Deoxyhypusine synthase
DOHH: Deoxyhypusine hydroxylase
GC7: N^1^-guanyl-1.7-diaminoheptane
1,3-DAP: 1,3-Diaminopropane
CHO: Chinese hamster ovary
A-tRNA: Aminoacyl tRNA
P-tRNA: Peptidyl tRNA
eEF1: Eukaryotic release factor 1

## Footnotes

1 The nomenclature has changed from IF-M2B*a *to eIF-4D to the current eIF5A.

2 As eIF5A mostly exists as the hypusinated form in cells or tissues, eIF5A usually denotes natural eIF5A containing hypusine. eIF5A may also designate the protein that is not modified. Only when a clear distinction of different forms is needed, the amino acid residue at the hypusination site is specified in parenthesis.

3 In mammals, there are two eIF5A genes, encoding highly conserved isoforms, eIF5A1 and eIF5A2, and both undergo hypusine modification. As eIF5A1 is the isoform predominantly expressed, eIF5A commonly represents eIF5A1. eIF5A can also be used to represent both forms collectively
